# Basic and Clinical Scientists Working Together—*Do We Make the Best of Both Worlds?*

**DOI:** 10.1007/s00223-025-01347-z

**Published:** 2025-02-14

**Authors:** Willem F. Lems, Athanasios D. Anastasilakis, Christina Møller Andreasen, Julien Paccou, Tim Rolvien, Michaela Tencerova, Jan Tuckermann, Maria P. Yavropoulou, Kent Søe

**Affiliations:** 1 Department of Rheumatology, Amsterdam UMC, Amsterdam, The Netherlands; 2 Department of Endocrinology, 424 Military General Hospital, Thessaloniki, Greece; 3 Pathology Research Unit, Department of Clinical Research, University of Southern Denmark, Campusvej 55, 5230 Odense M, Denmark; 4 Department of Pathology, Odense University Hospital, Odense, Denmark; 5 MABlab ULR 4490, Rheumatology Department, University of Lille, CHU Lille, F-59000 Lille, France; 6 Department of Trauma and Orthopaedic Surgery, Division of Orthopaedics, University Medical Center Hamburg-Eppendorf, Hamburg, Germany; 7 Laboratory of Molecular Physiology of Bone, Institute of Physiology of the Czech Academy of Sciences, Prague 4, Czech Republic; 8 Institute of Comparative Molecular Endocrinology (CME), Ulm University, Ulm, Germany; 9 First Department of Propaedeutic and Internal Medicine, Endocrinology Unit, LAIKO University Hospital, National and Kapodistrian University of Athens, Athens, Greece

**Keywords:** Basic scientist, Clinical scientist, Barriers, Systems medicine, Organisation, Culture

## Abstract

Musculoskeletal disorders, affecting as many as 1.3 billion people worldwide, are the leading cause of disability and impose a substantial health and socioeconomic burden. Despite the high prevalence of these conditions, translational research in this field is far from optimal, highlighting the need for stronger collaboration between basic and clinical scientists. This paper, authored by members of the basic and clinical action groups of the European Calcified Tissue Society (ECTS) and endorsed by the Board of the ECTS, examines the key barriers to effective translational research in musculoskeletal diseases, including clinician workload, differences in professional language and culture, physical distance between research sites, and insufficient interdisciplinary funding. Through interviews with eight institutional managers across five European countries, we observed that in some institutions, the collaboration between basic scientists and clinicians was regarded as no concern (but with room for improvement), and in most institutions it was recognised as a serious issue. We found consensus on the importance of collaboration yet identified discrepancies in the provision of structural and financial support. Based on these findings, we propose strategic initiatives to bridge the gap between basic and clinical research. Suggested measures include dedicated translational funding, integrated research facilities, collaborative scientific forums, strategic collaborations, establishment of physician-scientists, and, finally, bringing basic and clinical researchers together in the same building or even in a combined department. Notable successes, such as the development of the anti-osteoporotic drugs, romosozumab and denosumab, underscore the value of a coordinated approach and exemplify how shared insights between laboratory research and clinical practice can lead to impactful therapeutic advances. Moving forward, we advocate for institutional commitments to foster a robust translational research environment, as well as tailored funding initiatives to support such efforts. This paper serves as a call for discussion and action to enhance interdisciplinary cooperation to advance musculoskeletal medicine and improve outcomes for patients with debilitating musculoskeletal diseases.

## Introduction

Musculoskeletal disorders represent a growing global health problem. The World Health Organization estimates that 1.3 billion people worldwide suffer from musculoskeletal conditions, making them the leading cause of disability [1]. Musculoskeletal disorders include a variety of diseases of the bones (e.g. osteoporosis), muscles (e.g. sarcopenia), joints (e.g. osteoarthritis), and other tissues such as teeth, tendons, and ligaments. These conditions affect not only the elderly but also the young and include not only common, but also many rare diseases.

The complexity of musculoskeletal disorders necessitates a collaborative approach between basic and clinical researchers to develop and implement effective preventive, diagnostic, and therapeutic strategies. While good collaboration exists in some hospitals and universities between basic and clinical scientists, there is a clear need to improve interdisciplinary collaboration, as critical gaps remain between basic research and clinical application. This disconnection hampers the translation of laboratory discoveries into tangible clinical benefits for patients. Bridging this gap is important in musculoskeletal medicine, where the pathophysiology of individual disorders is intricate and multifaceted, involving genetic, biochemical, inflammatory, hormonal, and mechanical factors. Despite the high prevalence and immense burden arising from these diseases, funding for musculoskeletal research remains disproportionately low compared to other major health areas, such as oncology and the cardiovascular field [2]. This funding gap hinders substantially progress in understanding the underlying mechanisms and developing new therapies, exacerbating the challenges faced by patients and healthcare systems.

Osteoporosis alone affects globally over 200 million individuals [3], with one in three women and one in five men over the age of 50 experiencing osteoporotic fractures [4]. One notable success story exemplifying the power of integrated basic and clinical research is the development of anti-sclerostin antibodies (romosozumab) for the treatment of osteoporosis. The journey began in 2001 with the discovery of loss-of-function mutations in the SOST gene, encoding for sclerostin, as a cause for the rare high bone mass disorders called sclerosteosis and Van Buchem disease [5]. Subsequent molecular research identified the protein, sclerostin, as a key inhibitor of bone formation. Translating this knowledge into clinical practice, researchers developed romosozumab to inhibit sclerostin, thereby promoting bone formation and to some extent reduce bone resorption. Already in 2014, clinical trials proved the efficacy of romosozumab in increasing bone mineral density and reducing the risk of fractures in patients with osteoporosis compared to placebo [6], and in 2017, also compared to alendronate [7]. Today, romosozumab is approved in many countries as a novel anabolic treatment for postmenopausal osteoporosis, thus representing a big step forward for postmenopausal women at high fracture risk [8, 9]. Another success story was the discovery of RANKL, the subsequent identification of its role in osteoclast development, and function (e.g. identified using transgenic animal models), and finally the clinical use of denosumab [10, 11], an anti-RANKL antibody, for the treatment of not only osteoporosis, but also other bone diseases characterized by increased bone resorption [12].

Although there are currently several effective drugs available to treat osteoporosis, there is always room for new and sophisticated agents to reinforce this effort, while for other common musculoskeletal disorders, such as osteoarthritis and sarcopenia, not a single drug has yet been approved, underlining the unmet need for increased collaboration and innovation. Furthermore, the multiple single nucleotide polymorphism candidates from the UK Biobank studies associated with bone mineral density required basic research, often in animal models to provide the significance of the involvement of candidate genes and thus being potential drug targets in bone biology (e.g. [13]). Here, the contribution of novel and unknown human factors still remains to be proven. To make progress, it is imperative that researchers across the spectrum of musculoskeletal science collaborate more effectively. Therefore, we introduce this position paper, authored by members of the Basic Science and the Clinical Practice Action Groups of the European Calcified Tissue Society (ECTS), and approved by the ECTS Board. We hope that it will open up the discussion about promoting such collaborations. Our manuscript aims to identify major obstacles and to outline strategic recommendations for fostering collaboration between basic and clinical researchers within academia and the public hospitals. By highlighting the successes and identifying the barriers to effective collaboration, we seek to pave the way for innovative research and improved clinical outcomes for patients suffering from musculoskeletal disorders.

## What is the Problem?

Translation of important basic science data into clinical practice has long been a pursue of medicine worldwide [14]. A working model of two-way interaction between basic scientists and clinicians is of critical importance in modern medicine to promote optimal translation of basic research into the clinical setting and to maximize benefits to the community [15]. Despite its well-established validity in medical research and medical practice, interaction between clinical and basic scientists nowadays faces multiple challenges mostly due to differences in work priorities, work-time schedule, work language/culture, and perspectives among scientists.

In essence, translational research uses observations from basic science and patient settings to learn more about a disease and to potentially develop more efficient treatments. This discipline, translational research or “systems medicine”, is truly effective when clinicians, knowing the clinical problem and managing their patients, join forces with basic scientists trained to investigate cellular and molecular mechanisms [16, 17]. Although the biological relevance of a biomarker may be limited seen from the clinical perspective, its discovery can be fundamental in tailoring the next patient treatment strategy.

We believe that the musculoskeletal field needs more success stories to improve the treatment of musculoskeletal diseases, but we find that this will require a more dedicated and coordinated effort than we have seen in the past. The synergy between clinicians and basic scientists holds immense potential. Therefore, basic and clinical research should join forces to unite clinical and basic knowhow in order to improve musculoskeletal healthcare and accelerate the development of tailored precision medicine. However, it can only happen when an effective and dedicated communication as well as interaction between basic scientists and clinicians is achieved. In most centres, the actual situation is that this interaction faces significant barriers to reach the bench-to-bedside goals of translational medicine. Understanding the reasons for these problems is the first step towards overcoming these barriers [18].

## Barriers Seen from Clinical Scientists’ Perspective

### Barrier 1: Clinical Workload

From the clinicians’ perspective, a major issue is the work overload that often exhausts clinicians and hinders communication with basic scientists. A clinician is nurtured and trained under the notion that “patients come first”. Patient care certainly must have the highest priority! However, when physicians are overwhelmed with patient care responsibilities and increasing administrative tasks, this drains time and energy to engage in meaningful discussions or collaborations with basic scientists. Initiating a project in collaboration with basic scientists is time-consuming and demands extra time for meetings to exchange ideas and specify the ways to materialize them. The lack of availability impedes the exchange of ideas, data, and research findings between the two groups. Acknowledging and addressing work overload in the healthcare setting is essential for promoting effective communication and collaboration between clinicians and basic scientists. Moreover, the urgency and immediacy of clinical work may not align with the more methodical and exploratory nature of basic research, creating challenges in finding common ground and fostering productive communication that could ultimately lead to advancements in medical research and patient care.

### Barrier 2: Lack of Bi-directional Understanding (Language and Culture)

Another important barrier is the lack of a “common” language between the two groups of scientists. The terminology and methodology of basic research differ substantially from the one that the clinicians are accustomed to. This often deters clinicians from coming in touch with basic scientists. Clinical scientists often focus on “real-world” applications and patient outcomes, while basic scientists may be more concerned with fundamental mechanisms and theoretical frameworks. Therefore, errors may cause irreparable damage in the clinic, but are inevitable manifestations of the creative process in the lab. This difference in perspective can lead to miscommunication and misunderstandings when collaborating on research projects. Clinicians are often concerned that basic scientists cannot fully understand the challenges of clinical research. In fact, many physicians are not interested in research regarding questions that they feel are unlikely to change clinical practice, at least in the short term [19].

In addition, experienced clinicians are accustomed to relying on clinically oriented journals and articles when analysing patient cases [15], while they do not usually seek basic science knowledge beyond the underlying mechanisms that will help them understand and efficiently treat these cases [20–22]. For a clinical scientist, to successfully move between the fields of scientific research and clinical medicine is quite challenging. The inherent danger of such a quest is that by trying to be good at both laboratory research and patient care, you end up failing at both. In this case, basic scientists may be concerned about the depth of a clinician’s scientific knowledge, while clinical colleagues may question his or her clinical understanding [19]. This scepticism, which may come from both groups, may ultimately hinder clinicians’ willingness to collaborate with basic scientists and work on basic research projects.

### Barrier 3: Economical Aspects—Organization

If a clinician spends more time and energy in the lab, he/she will spend less time in the clinical department, a behaviour that may not be well perceived by clinical colleagues or the department head. Furthermore, on many occasions, clinicians are compensated according to the number of patients they treat while collaboration with basic scientists is not expected to be compensated; on the contrary, extra time and effort is required to be funded for a common project.

A physical distance between basic research facilities and hospitals, which is common, can be an obstacle in the collaboration between clinical and basic scientists in several ways. Physical distance creates logistical challenges, making it difficult for researchers to leave their “comfort zone” and meet in person, share resources, and exchange ideas effectively. Face-to-face interactions are often critical for building relationships, fostering trust, and facilitating interdisciplinary collaborations. In addition to distance, working in another department with another hierarchy is also a barrier.

## Barriers Seen from Basic Scientists’ Perspective

From the point of view of a basic researcher, there are several critical factors that contribute to scientist-clinician group barriers.

### Barrier 1: Different Priorities

The priorities in setting up basic experiments and clinical studies differ considerably. These differences usually evolve into a critical obstacle when trying to integrate basic research questions into clinical studies. For instance, the demands for patients’ biological samples from both sides may be impossible to harmonize and integrate into patients’ clinical visits—or even to get patients to give their consent—for that matter.

### Barrier 2: Lack of Communication, Trust, and Understanding

Many basic scientists experience that fruitful communication between basic and clinical scientists is often hindered by the lack of a common language. This often leads to misunderstandings and frustrations and in the end-effect prevents a common understanding of the need and the purpose of studies both from basic and clinical scientists’ point of view. Another element contributing to this barrier is time for the discussion about the design of studies, downstream molecular analyses, and the outcome of the studies. The motivation for basic scientists to a research question is often to start with the question “why?”, while for clinicians it may rather be a question of “how and when”. The basic scientist’s curiosity is driven by the knowledge-gap that leads them into deeper basic molecular research, while clinicians are driven by the goal to help the patient by offering realistic and suitable treatments as soon as possible. This often leads to the misconception that basic scientists concentrate on rather irrelevant details, distant from the “real-world”, which may unnecessarily prolong the studies. However, these detailed investigations may contribute to a more detailed understanding of the mechanisms central for a given treatment’s efficacy or bring solutions to why a given treatment is not effective on all patients.

Finally, in some countries, medical departments are much more hierarchically organized than basic science laboratories; in this case, clinicians may be experienced by basic researchers as arrogant, which simply may be a prejudice by the basic researcher, which is, however, challenging communication on equal footing.

To achieve a common ground for combining both clinical and basic research results to make a change for the patient, this demands trust and understanding. These on the other hand can only be achieved by dedicating time from both parties to reach common ground. Precisely this could be a major obstacle for a successful collaboration.

### Barrier 3: Physical Distance

Rather long distances between basic research institutions and hospitals make it even harder to overcome the obstacles listed above, as time is limited for all. Therefore, the two worlds often only meet in formal meetings, but the best interactions are often obtained when taking place informally, e.g. meeting at lunch or the coffee machine. Meetings that are more informal could serve to create a trust-based relationship, something that is critical if research should lead to important discoveries. A distance between these two “worlds” nurtures the perception of “them” and “us”, which is an obstacle for a trustful interaction.

### Barrier 4: Research Funding

If common grounds have been obtained for a coordinated research effort between basic and clinical scientists, another hindrance for its success is the limitations in research grants that specifically support research uniting basic and clinical scientists. However, the significance of such grants has garnered increased attention in recent years, as they are seen as important for fostering improved translational research collaborations in the future.

## How Does the Management of Hospitals and Universities See the Problem?

Looking at the problems described from the perspective of basic and clinical scientists, there are indeed commonalities. Both parties (basic and clinical scientists) agree that it is important to better utilize the unique skills and expertise of both worlds. Both parties agree that the major obstacles are

- Lack of a common language
- Lack of bidirectional understanding
- Lack of time needed to dedicate in fruitful interactions
- Different locations—long distances
- Lack of funding opportunities

To be successful in overcoming such obstacles, it is critical to have the support from the management of not only individual departments, but more importantly the management of the institutions.

Therefore, we decided to hear the perspective of the management in our own institutions on this matter. We (all authors) agreed on the wording of the questions to standardize the procedure. We contacted the managers, and all were asked the same questions. Some managers chose to reply in writing while others gave their replies through interviews. The questions asked were as follows:

1. Do you think there is a lack of interaction and collaboration between basic scientists and clinicians at your institution? Yes/No?
2. If YES, do you think that a lack of interaction between basic and clinical scientists is a concern? Why/why not
3. Is this an issue that you give priority at your institution? Why?/why not?
4. Do you have existing initiatives to facilitate interaction between basic and clinical scientists? Could you mention some examples? (Infrastructure, recruitment strategies, management, meetings, conferences, funding etc.)
5. Do you have plans for facilitating interaction between basic and clinical scientists? How?
  (A) Projects/strategies etc.
  (B) Financial support?
6. Do you think there are sufficient good conditions by governmental or by funding programmes to foster translational research?
7. What kind of benefits do you expect from practical interaction between basic and clinical scientists?
8. What are the limitations to implement better interaction between basic and clinical scientists?

We have kept the responses from the institutions anonymous, because it is not important who answered what, but rather what are the different views on and solutions to the lack of collaboration between clinical and basic scientists. To summarize the responses, so that they can be compared, we have grouped the responses in Table 1.

**Table 1:** Summary of the answers from the management of the authors’ institutions regarding the clinical-basic scientist interactions

Institution	Concern? Yes/no	Importance	Initiatives	Problems
Institution 1	Yes, major concern	Effectively tackle medical challenges Pioneer new therapies Improve the advancement and knowledge of scientists	> 10% of academic faculty should be basic scientists Joint conferences Research infrastructure for services in personalised medicine External research programmes Interaction of medicine with philosophy or physics through educational programmes and seminars	Lack of funding for promoting interdisciplinary research Lack of staff at universities Historical competition between disciplines However, younger generations are more collaborative
Institution 2	No, not a concern	Improved funding opportunities New diagnostic and therapeutic tools	None	Lack of funding for both clinical and basic research
Institution 3	No, not a concern	It is our mission as a university medical centre Translate scientific achievements into clinical practice Opportunity to gain access to patient cohorts	Funding joint basic/clinical projects in specific research areas Interdisciplinary networking through PhD-School PhD candidates must have interdisciplinary mentors Scientific speed-dating	Insecure and temporary employment of basic scientists Strict regulation of medical doctors’ clinical duties
Institution 4	No, but room for improvement	Improved quality of basic science Improved track records of MDs Improved understanding of science amongst MDs Improved access to funding	Facilitate joint meetings Funding of PhD-students Support scientists in obtaining funding	Time Not enough attractive funding Some people need to work more
Institution 5	Yes, it is a concern	Lack of contacts/communication prevents research progress Better methodology will impact both science and industry	There are no initiatives in action Attempts are made, but are prevented by resistance from a significant proportion of both clinicians and basic scientists Created a centre for interdisciplinary research and innovation	Everyday routine workload makes such a concern a luxury Underestimating the role of basic scientist by clinicians and vice versa Lack of funding
Institution 6	Yes, it is a concern	Delay between discovery and implementation into clinical practice hinders patient benefit Better and more precise research that leads to patient tailored treatment	Management Forums between hospital and several faculties at the university The hospital allocates internal funds to create interdisciplinary elite research environments Financial support for grant writers Encourage supervision of PhD-students by interdisciplinary mentors Common research strategy with the university focussing on excellence and interdisciplinary research Heads of research are 4 times a year asked to report to a reflection team on the progress in fulfilling the research strategy	Personality, specialists are individualists and thrive on individuality—hence favour own concepts—hinder alternative solutions e.g. including basic science Reluctance of clinicians to venture beyond their comfort zone Physical distance between institutions Medical students lack adequate scientific training in research Experienced doctors may not sufficiently inspire the younger generation to pursue research endeavours
Institution 7	No, but more collaboration would be even better	‘Silo thinking’ would lead to a lack of development Only through cross-collaboration, the relevant questions can be identified	Relocation to new facilities where hospital and university are under one roof The university funds interdisciplinary frontline and elite research environments Clinical Professors have 50% of their time dedicated to research Arranges ‘speed-dates’ in an inspirational forum to keep research leaders on their toes, ensuring progress on interdisciplinary research Recruitment of basic researchers with a clinical focus is common practice (funded by the hospital) 10% of all research employees must have another training than health professional Medical students can choose either a research track or a clinical track Evidence-based medicine is in focus Created physical spaces for spontaneous and informal gatherings between different specialties Respect the need for also ‘eccentric’ personalities	Hospital department leaders are gate keepers for prioritizing funding and time for research positions Staff shortages causes projects to fail because they are burdened with clinical responsibility Some people think it is enough to focus on themselves Physical distance between institutions National foundations express reluctance to genuinely support translational research
Institution 8	Yes, it is a concern	Increasing economic pressure and process optimisation in the clinical setting with decreasing research time Increasing complexity of scientific methods Deduction of clinically meaningful hypotheses Improved quality of clinical studies Combination of clinical outcome and mechanistic studies Benefits for the individual patient and society	At the Medical Faculty we support clinical scientists by a structured programme Have establishment a basic and advanced Clinical Scientist Programme Introduction of a Scientific Consultant Physician The establishment of a Clinician Scientist Academy with regular meetings Medical Scientist Programme for experienced postdocs (at least 3 years after PhD) in the field of molecular medicine Plan to integrate clinician scientists in collaborative research efforts Continue the financial support by the Medical Faculty for the clinician scientist programmes	Improvement of translational research is necessary but currently significantly underfunded Time limitations in the clinical setting Research-independent career paths

We received replies from eight out of 10 institutions (University of Southern Denmark, Odense University Hospital, Ulm University, University Medical Center Hamburg-Eppendorf, Aristotle University of Thessaloniki, Institute of Physiology of the Czech Academy of Sciences, Lille University Hospital, and National and Kapodistrian University of Athens situated in five different European countries (Greece, Denmark, France, Czech Republic, and Germany)). Despite clear differences in the level of detail in the replies, there is a considerable overlap in the key messages from these eight institutions, which can be summarized as follows:

1. Concern: four out of eight institutions do not think it is a concern at their institutions.
  This may be because they find that they have already done enough or that it is not of their concern.
2. Importance: the managements of all eight institutions agree that collaboration between basic and clinical scientists is favourable and will bring more scientific success.
3. Initiatives: the replies to this question differ somewhat, but most managements seem to be satisfied with the initiatives they have taken to facilitate collaboration between basic and clinical scientists, such as collaborative scientific meetings and collaborative education. Some institutions clearly have more elaborated and coordinated plans than others do. However, some also point out that they have tried to implement initiatives, but experienced that the scientists, at least as perceived by the management, did not want to engage.
4. Problem: with few exceptions, there is not much structural or financial support to facilitate successful collaboration between basic and clinical scientists. However, various issues that relate to time and employment conditions are seen as a problem across institutions. Some also highlight the difficulties in obtaining synergy because there is a lack of communication and understanding across the disciplines and institutions.

## Concluding Remarks

Even though there have been major advances in biomedical research over the last decades, our survey of universities and hospitals in five European countries has shown that the collaboration between basic and clinical scientists is still far from being optimal in many organizations. Although successful collaboration is desirable, because it can speed up the success rate in the field, both basic and clinical researchers experience limitations in their daily work, which prevent them from efficiently interacting with each other. The research agenda in musculoskeletal diseases is still very long i.e. valuable treatment options in osteoarthritis and sarcopenia, personalised medicine, drugs that have durable bone-anabolic effects, and maybe even drugs that will favour a reestablishment of coupling between bone resorption and formation. To have success in this regard, we urgently need a stimulating translational collaboration in the field.

Our group, comprised of both basic and clinical scientists, has summarized the main barriers based on personal experience including (i) work overload (e.g. urgent clinical tasks), (ii) lack of common language and culture, (iii) infrastructure issues in the organizations (e.g. different buildings for basic and clinical research that are distant from each other) and (iv) lack of targeted funding of collaboration projects that play a critical role.

We also invited the opinion of managers/board members of our hospitals/universities, demonstrating a remarkable overlap in their responses. The interviewed managers collectively acknowledge the fact that collaboration between basic and clinical scientists is of paramount importance and will bring more scientific progress, and they all agree that regular collaborative scientific meetings and shared education programmes may be helpful. However, structural and/or financial support for translational collaboration between basic and clinical scientists is for most of them not (yet) on the top of their agenda.

**Fig. 1:**
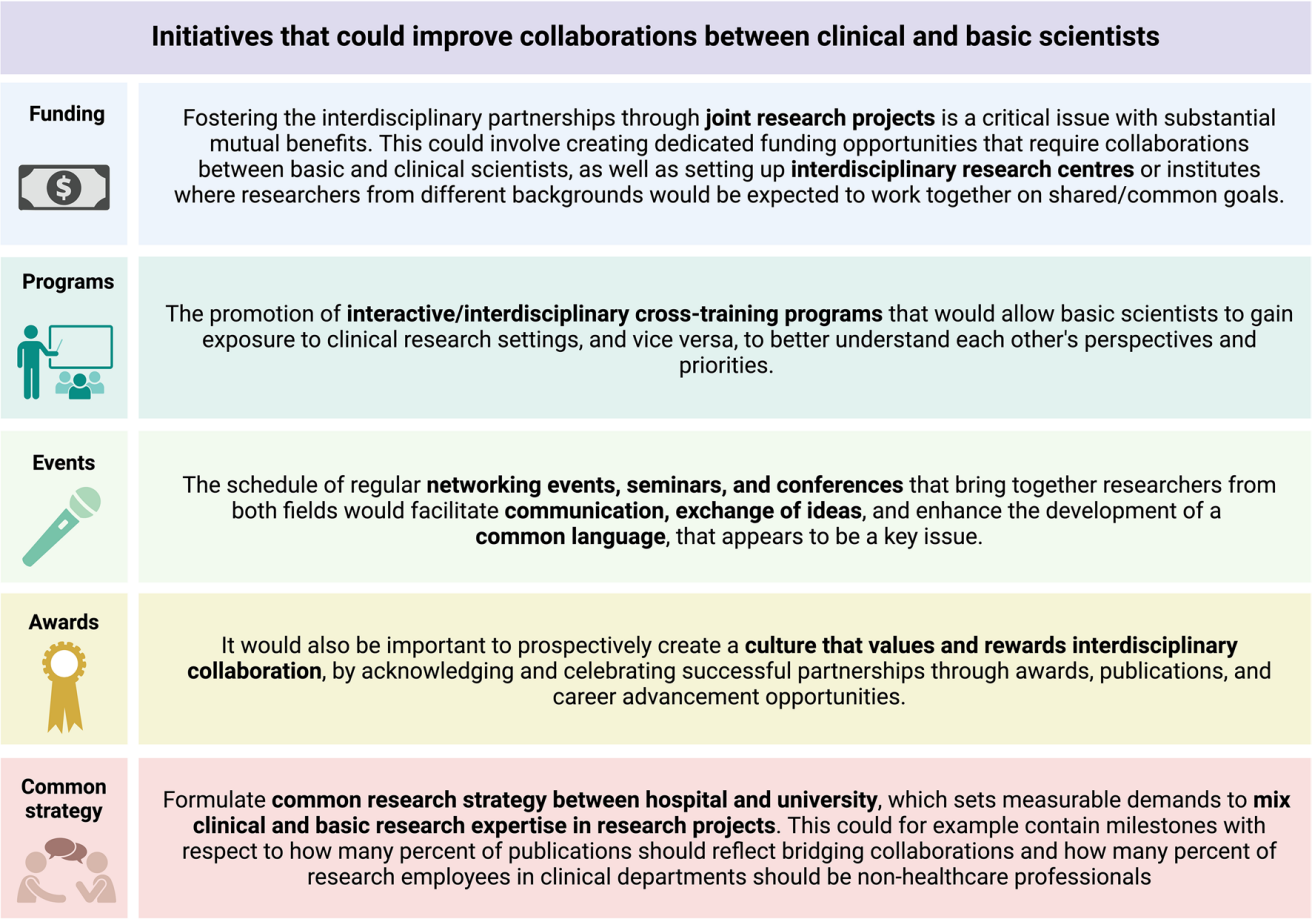
Initiatives that could improve collaborations between clinical and basic scientists. Created with Biorender.com

By analysing the collected responses and interviews of both basic and clinical scientists as well as managers, we have summarized below some suggested initiatives that would be perceived as effective from the scientists’ perspective (Fig. 1).

**Fig. 2:**
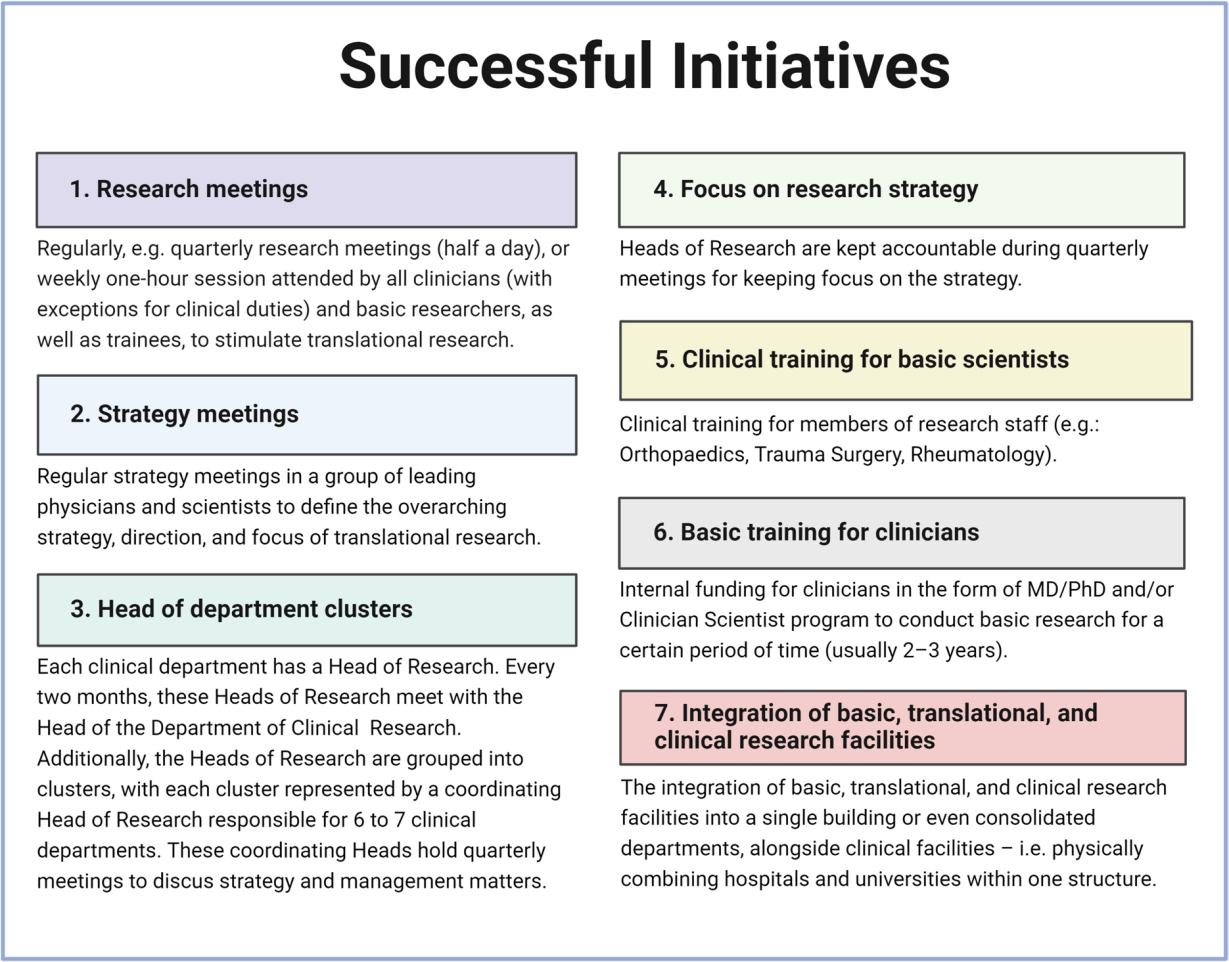
Examples of successful initiatives as perceived by the authors. Created with Biorender.com

Apart from the suggested initiatives presented in Fig. 1, the authors have also evaluated what is perceived as successful and effective existing initiatives at the home institutions of the authors. These examples are presented in a simplified manner in Fig. 2. One of these solutions to the translational problem in musculoskeletal diseases is to develop, promote, and expand the pool of clinician scientists, for example, through dedicated MD/PhD or clinician scientist programmes. This would be favourable to facilitate a more direct communication with basic scientists and thereby bring ideas from bench to bedside and vice versa. However, over the years, these experts have become rare, and with the enormous progress in both basic techniques, clinical opportunities, as well as work load they may be even more rare in the near future. Precisely therefore, creating more opportunities for basic and clinical scientists to work together will help to bridge the gap between these disciplines and foster a more integrated approach to biomedical research.

One may argue that our manuscript is too focussed on universities and hospitals. The situation seems to be different in pharmaceutical industries; based on a clear and strict hierarchy under one leader, all departments are working together on one theme, the development of a new drug such as the remarkably rapid and successful development of e.g. denosumab and romosozumab (as highlighted in the Introduction). This only highlights the strength of unifying, promoting, and coordinating the efforts of basic and clinical scientists. Although a similar coordinated and structured effort cannot be implemented one-to-one in the research efforts within academia and hospitals, it may be advisable to seek inspiration from the pharmaceutical industry when making efforts to optimise research efforts between academia and hospitals.

Finally, when scientific organisations such as ECTS, ASBMR (American Society of Bone and Mineral Research), and other national and international organisations plan meetings and conferences, it could be favourable to consider that participants may not always be limited to basic or clinical interests, but may actually be interested and engaged in both. When considering this, scientific conferences and meetings may be able to further stimulate coordinated research efforts between basic and clinical scientists.

In conclusion, this manuscript points out on the missing interactions between basic and clinical scientists and highlights the importance of fostering interdisciplinary collaboration between basic and clinical scientists in order to drive innovation and advances in musculoskeletal medicine. Today, more than ever, the scientific community recognizes that the future of biomedical research lies in the seamless integration of basic and clinical science, and that by working together, basic and clinical scientists can unlock new possibilities and make meaningful strides towards improving human health and well-being through personalised medicine and treatments that are more effective. We hope that our perspective will contribute to future discussions and activities to strengthen translational collaboration between basic and clinical science and to promote viable collaborative research strategies among hospitals, universities, and research institutions.
